# The Effect of Coronary Angiography Timing on Cardiac Surgery Associated Acute Kidney Injury Incidence and Prognosis

**DOI:** 10.3389/fmed.2021.619210

**Published:** 2021-04-15

**Authors:** Kang Liu, Meiyuan Li, Li Li, Buyun Wu, Xueqiang Xu, Yifei Ge, Huijuan Mao, Changying Xing

**Affiliations:** Department of Nephrology, The First Affiliated Hospital of Nanjing Medical University (Jiangsu Province Hospital), Nanjing, China

**Keywords:** coronary angiography, cardiac surgery, acute kidney injury, contrast media, interval time

## Abstract

**Introduction:** Acute kidney injury has been identified as a common complication of cardiac surgery. To date, the effect of the time interval from coronary angiography to cardiac surgery on postoperative acute kidney injury is still controversial. The aim of this study was to investigate the relationship between the timing of coronary angiography and cardiac surgery associated acute kidney injury.

**Methods:** Eight hundred thirteen patients who underwent coronary angiography and cardiac surgery successively from January 2017 to December 2018 were included in this retrospective cohort study. We applied multivariate logistic regression, propensity score analysis, and subgroup analysis to evaluate the association between the time interval and postoperative acute kidney injury incidence and prognosis. Meta-analysis was conducted to verify the results.

**Results:** The overall incidence of the cardiac surgery associated acute kidney injury was 28.8%. Age (OR = 1.046, 95%CI: 1.017–1.075), cardiopulmonary bypass (OR = 3.439, 95%CI: 1.316–8.986) and diabetes (OR = 2.522, 95%CI: 1.439–4.417) were found to be independent risk factors of postoperative acute kidney injury in multivariate logistic regression and propensity score analysis. Undergoing cardiac surgery within 7 days after coronary angiography was not associated with increased incidence of postoperative acute kidney injury or worse prognosis. Meta-analysis obtained consistent results.

**Conclusions:** The time interval shorter than 7 days had no influence on cardiac surgery associated acute kidney injury incidence and prognosis. The decision of delaying the surgery should be made after comprehensive evaluation of the patient.

## Introduction

Cardiac surgery associated acute kidney injury (CSA-AKI) is a serious and common complication after cardiac surgery. Because of variable definitions, the incidence of CSA-AKI ranges from 9 to 40%, with 1–7% of cases requiring dialysis. CSA-AKI is one of the strongest predictors of mortality, and can delay recovery in up to 30% of patients (1–3). The etiology and pathophysiology of AKI after cardiac surgery are complex and not fully understood. It is well-established that the underlying mechanisms include oxidative stress, ischemia-reperfusion injury, inflammation, and nephrotoxins (4). As there are not sufficient effective interventions to improve the prognosis, early identification, and modification of risk factors are especially critical.

Cardiac surgical procedures often follow diagnostic coronary angiography (CAG). However, contrast agent is a recognized risk factor for contrast-induced acute kidney injury (CI-AKI), which has an incidence of 7–10% (5, 6). To date, it is uncertain whether coronary angiography prior to cardiac surgery may enhance the risk of CSA-AKI. According to previous literatures, many predisposing factors are associated with the CSA-AKI, include age, diabetes, cardiac insufficiency, pre-existing kidney disease, and cardiopulmonary bypass (CPB) (7–10). One of the great challenges is that most of the risk factors are inherent and unmodifiable. But the time interval from CAG to cardiac surgery can to some extent be adjusted by clinicians. Del Duca et al. found that performing cardiac surgery within 5 days after CAG increased the risk of postoperative AKI (9), Although this was contradicted by another report (10). In other words, the optimal timing of surgery following angiography remains controversial.

To clarify relationship between the timing of surgery after CAG and CSA-AKI, we conducted a study in a large cohort of Chinese patients. In the meanwhile, we performed a comprehensive meta-analysis to validate our findings.

## Materials and Methods

### Study Population and Perioperative Management

All 2,112 adult patients undergoing invasive CAG and elective cardiac surgery at Jiangsu province hospital from January 1st, 2017 to December 31th, 2018 were screened for this retrospective cohort study. Patients were excluded if they met the following criteria: (1) <18 years old; (2) received renal replacement therapy (RRT) before the surgery; (3) had a time interval from CAG to surgery >30 days; (4) underwent percutaneous coronary intervention; (5) underwent emergency or salvage surgery; (6) had vital signs that were not stable (e.g., in the case of shock) or required mechanical ventilation before surgery; and (7) had missing data. Ultimately, a total of 813 patients were included in the analysis. All data were retrieved from the hospital information system (HIS), which includes electrical medical record (EMR), laboratory information system (LIS), anesthesia information management system (AIMS), and nursing information system (NIS). The institutional review board and Ethics Committee of Jiangsu province hospital approved this study.

Perioperative management, CAG procedure, anesthetic, and surgical techniques were standardized for all patients. Surgical options and the use of cardiopulmonary bypass were decided by a same professional team of cardiac surgeons. All enrolled patients were advised to avoid potentially nephrotoxic drugs during hospitalization and were routinely given adequate hydration with intravenous normal saline at a rate of 1 ml/kg/h for 12 h after angiography. All patients received iso-osmolar contrast media (CM) at an iodine concentration of 320 mg/ml (Iodixanol, Visipaque®, GE Healthcare, Ireland) and amount of CM was determined in light of each patient's weight. For patients with baseline serum creatinine (SCr) >133 umol/L, the amount of CM was restricted, and N-acetylcysteine (1,200 mg) was administered every 12 h, 1 day before and after angiography.

### End Points and Definitions

The primary endpoint was the incidence of cardiac surgery associated acute kidney injury. CSA-AKI was defined according to Kidney Disease Improving Global Outcome (KDIGO) Clinical Practice Guidelines: increase in SCr by ≥26.5 umol/L within 48 h; or increase in SCr to ≥1.5 times baseline that is known or presumed to have occurred within the prior 7 days (11). Urinary output criteria in defining AKI were not considered as there was frequent use of diuretics in perioperative period, which would make it unreliable. Patients with AKI were staged on the basis of KDIGO classification. Severe AKI was defined as stage 2 or 3 AKI. Baseline laboratory values were collected at admission or immediately before surgery when CAG was performed on the same operation day. Estimated creatinine clearance (eGFR) was calculated using the Chronic Kidney Disease Epidemiology Collaboration (CKD-EPI) equation (12).

### Statistical Analysis

Continuous variables were examined for normal distribution by the Kolmogorov-Smirnov test and summarized as either mean ± standard deviation or medians with interquartile range if the distribution of variables was skewed. Unpaired Student *t*-test or Mann-Whitney *U*-test were used to compare between groups. With regard to dichotomous variables, between-group differences were tested by Pearson chi-square or Fisher exact test.

We collected five types of variables that could potentially influence the occurrence of CSA-AKI: demographic information, previous history, preoperative data, intraoperative situation, and postoperative outcome. Basic patient information included age, gender, smoking history, and significant comorbidities such as hypertension, diabetes mellitus, cerebrovascular disease (CVD), anemia, and chronic obstructive pulmonary disease (COPD). Kidney and cardiac functions were assessed by SCr, eGFR, New York Heart Association (NYHA) classification, left ventricular ejection fractions (LVEF), and pulmonary arterial pressure (PAP). Perioperative parameters included important laboratory tests, American Society of Anesthesiologists (ASA) grade, operating time, CPB time, cross-clamp time, and fluid infusion.

First, the interval between CAG and cardiac surgery was deemed as a continuous variable and modeled as restricted cubic splines with 4 data-driven knot locations to account for the possible non-linear regression relationship with CSA-AKI. According to the restricted cubic splines and previous reports, we divided the patients into two time interval groups using 7 days as the cutoff value. We defined the operation on the same day of the CAG as interval 0. Subsequently, univariable analysis for the occurrence of CSA-AKI was carried out and statistically significant variables were tested in an adjusted logistic regression model. Calibration and discrimination of the multivariable model were evaluated by the Hosmer-Lemeshow goodness-of-fit test and the area under the receiver operating characteristic curve, respectively. In order to verify the stability of the adjusted logistic regression model, the time interval was reincorporated into the regression model as a continuous or multiple categorical variable. Furthermore, to control for selection bias related to covariates and make the results more reliable, a propensity score analysis was performed. The method generated a propensity score within 0 to 1 and the patients in ≤ 7 days group were matched 1:1 to patients in >7 days group, using the nearest neighbors matching algorithm. The propensity score was created based on variables that were same as those that were adjusted in the multivariate logistic regression. Absolute standardized differences showed by love plot were estimated to evaluate the prematch imbalance and postmatch balance and the differences <10% were considered inconsequential (13, 14). Lastly, subgroup analyses stratified by CPB, age, gender, diabetes mellitus, operation type, cardiac function, and kidney function were also conducted.

### Meta-Analysis

A meta-analysis was further performed to validate the association between the interval time and CSA-AKI. We searched the electronic databases PubMed, Embase, and the Cochrane Library up to February 29th, 2020 using the keywords: “catheterization or angiography or percutaneous coronary intervention” and “cardiac surgery” and “acute kidney injury or AKI or renal failure” without restrictions on the baseline conditions of study objects. Included studies met the following criteria: (1) the study investigated the risk of AKI and 7-day interval between CAG and cardiac surgery; (2) the study provided sufficient information to calculate odds ratios (ORs) and 95% confidence intervals (CIs); and (3) the study was a randomized controlled trial, cohort or case-control study. Two authors (M Li and K Liu) extracted the following data: first author, publication year, country, sample size, gender ratio, NYHA, operation type, AKI definition, and the number of patients suffering from AKI, severe AKI, RRT and death. Disagreements were resolved by discussion or consensus with a third reviewer (L Li). The study contained detailed data of patients undergoing on-pump or off-pump cardiac surgery was regarded as two separate studies. The quality of studies was assessed by the Newcastle-Ottawa scale (NOS) and those with scores of six stars or greater were considered to be of high-quality studies (15).

Summary ORs with their corresponding 95% CIs were used to assess the strength of the association. Between-studies heterogeneity was evaluated by the chi-square-based *Q*-test and *P* < 0.10 indicated the existence of heterogeneity. The pooled OR estimate of each study was calculated with the random-effects models using the DerSimonian and Laird method when these studies were heterogeneous (16). Sensitivity analysis was performed to determine whether a particular study or studies would result in heterogeneity and assess the stability of the results. Begg's funnel plot and Egger's regression test were applied to examine publication bias. All tests in this study were two-sided with the alpha level set at 0.05 for statistical significance. Statistical analyses were performed with Stata, version 15.1 (StataCorp LP, College Station, TX, USA).

## Results

### Baseline Characteristics

The baseline demographics, clinical characteristics, intraoperative, and postoperative variables of the 813 included patients are shown in Table 1. The mean age was 62.5 ± 8.9 years and there were 493 (60.6%) males. The overall incidence of the CSA-AKI was 28.8%. The majority of patients suffered from stage 1 AKI (62.4%) and 27 required dialysis (3.3%). There was no significant difference in AKI incidence between two groups. Compared to patients who underwent cardiac surgery after 7 days, those who had surgery within 7 days of CAG had fewer comorbidities such as hypertension, diabetes mellitus, and cerebrovascular disease and had a higher ejection fraction, albumin level, and hematocrit value. In terms of surgical procedure, the median operation time, blood loss volume, ultrafiltration volume were higher and the proportions of CPB, platelet infusion were increased in the shorter interval ( ≤ 7 days) cohort. Nonetheless, the number of patients requiring RRT and in-hospital mortality rate were similar between the two groups.

**Table 1:** Baseline and perioperative characteristics of the patients stratified by the CAG interval.

**Variables**	**Prematch**			**Postmatch**		
	**>7days (*n* = 367)**	**≦7days (*n* = 446)**	***P***	**>7days (*n* = 216)**	**≦7days (*n* = 216)**	***P***
**Demographic data**						
Age (year)	63.6 ± 8.8	61.6 ± 8.8	** <0.001**	62.9 ± 8.5	62.9 ± 8.8	0.791
Male	242 (65.9)	251 (56.3)	**0.005**	136 (63.0)	141 (65.3)	0.616
**Previous history**						
Smoking	131 (64.3)	131 (70.6)	0.055	73 (33.8)	79 (36.6)	0.546
Hypertension	212 (57.8)	200 (44.8)	** <0.001**	121 (56.0)	114 (52.8)	0.499
Diabetes mellitus	87 (23.7)	55 (12.3)	** <0.001**	40 (18.5)	41 (19.0)	0.902
COPD	5 (1.4)	8 (1.8)	0.623	1 (0.5)	4 (1.85)	0.372
Cerebrovascular disease	53 (14.5)	42 (9.4)	**0.026**	32 (14.9)	24 (11.1)	0.244
Anemia	18 (4.9)	24 (5.4)	0.760	10 (4.6)	14 (6.5)	0.401
**Kidney and cardiac status**						
Serum creatinine (umol/L)	73.7 (61.4, 84.1)	70.3 (59.6, 81.5)	**0.029**	74.0 (59.2, 84.7)	73.2 (61.7, 82.9)	0.875
eGFR (ml/min/1.73m^2^)	90.9 (79.2, 101.9)	93.0 (80.6, 102.4)	0.102	89.6 (78.0, 101.4)	91.0 (80.4, 101.1)	0.762
NYHA class I-II	226 (61.6)	264 (59.2)	Ref	139 (64.3)	126 (58.3)	Ref
NYHA class III-IV	140 (38.4)	182 (40.8)	0.459	77 (35.7)	90 (41.7)	0.199
Ejection fraction (%)	62.1 (53.7, 64.6)	62.7 (58.7, 65.1)	**0.006**	62.7 (57.9, 65.2)	62.2 (55.9, 64.8)	0.229
PAP (mmHg)	33 (27, 41)	33 (28, 40)	0.726	34 (28, 41)	31 (27, 40)	0.255
**Preoperative data**						
Hemoglobin (g/L)	131.5 ± 16.1	131.2 ± 16.6	0.800	133.0 ± 16.6	131.1 ± 16.9	0.397
Albumin (g/L)	38.3 ± 4.00	39.2 ± 3.8	**0.004**	39.0 ± 3.9	38.8 ± 3.8	0.737
Hematocrit (%)	37.4 ± 6.6	38.9 ± 7.0	** <0.001**	38.4 ± 6.6	38.2 ± 7.6	0.808
**Intraoperative data**						
**Operation type**						
Isolated CABG	197 (53.7)	94 (21.1)	**<0.001**	84 (38.9)	83 (38.4)	1.000
Isolated valve	112 (30.5)	251 (56.3)		88 (40.7)	89 (41.2)	
CABG+valve	39 (10.6)	65 (14.6)		28 (13.0)	28 (13.0)	
Other types	19 (5.2)	36 (8.0)		16 (7.4)	16 (7.4)	
ASA Grading	3 (3, 3)	3 (3, 3)	**0.006**	3 (3, 3)	3 (3, 3)	0.909
CPB	177 (48.2)	354 (79.4)	** <0.001**	141 (65.3)	135 (62.5)	0.548
Operating time (min)	257 (217, 336)	277 (233, 343)	**0.003**	276 (225, 357)	264 (220, 330)	0.235
CPB time (min)	143 (117, 190)	141 (111, 178)	0.301	140 (117, 190)	143 (110, 188)	0.694
ACC time (min)	103 (76, 139)	103 (76, 134)	0.933	105 (78, 142)	108 (75, 138)	0.832
**Blood transfusion**						
Red blood cells	317 (86.4)	370 (83.0)	0.180	185 (85.6)	182 (84.3)	0.686
Plasma	50 (13.6)	63 (14.1)	0.837	31 (14.3)	27 (12.5)	0.572
Platelets	162 (44.1)	321 (72.0)	** <0.001**	127 (58.8)	125 (57.9)	0.845
**Fluid balance**						
Blood loss (ml)	500 (500, 800)	700 (500, 1,000)	** <0.001**	600 (500, 800)	600 (500, 800)	0.828
Ultrafiltration (ml)	2,000 (0, 2,500)	2,100 (1,500, 2,700)	** <0.001**	2,000 (1,300, 2,600)	2,000 (1,000, 2,500)	0.776
**Postoperative data**						
Acute kidney injury	104 (28.3)	130 (29.2)	0.800	67 (31.0)	67 (31.0)	1.000
KDIGO stage 1	65 (17.7)	81 (18.2)		46 (21.3)	40 (18.5)	
KDIGO stage 2	22 (6.0)	28 (6.3)		17 (7.9)	16 (7.4)	
KDIGO stage 3	17 (4.6)	21 (4.7)		4 (1.8)	11 (5.1)	
RRT	13 (3.5)	14 (3.1)	0.757	2 (0.9)	7 (3.2)	0.175
In-hospital mortality	15 (4.1)	25 (5.6)	0.319	7 (3.2)	15 (6.9)	0.080
Length of hospital stay (day)	23 (19, 31)	19 (16, 23)	** <0.001**	25 (19, 31)	18 (15, 22)	** <0.001**

*eGFR, estimated glomerular filtration rate (CKD-EPI formula); NYHA, New York Heart Association; PAP, pulmonary artery pressure; CABG, coronary artery bypass grafting; CPB, cardiopulmonary bypass; ACC, Aortic cross clamp; KDIGO, Kidney Disease: Improving Global Outcomes; RRT, renal replacement therapy*.

*Bold characters are used to express P-values that are statistically significant (< 0.05)*.

### Association Between the Time Interval and CSA-AKI

Univariate logistic regression analysis was performed to identify the predictors of CSA-AKI (Table 2). Older age, diabetes, cerebrovascular disease, worse renal function, lower levels of hemoglobin and albumin, CPB, prolonged operation time, large bleeding volume, and ultrafiltration volume might increase the hazard of CSA-AKI. However, we failed to observe any correlation between the time interval and CSA-AKI. It is worth noting that AKI had a marked impact on patient prognosis, resulting in longer hospital stays and higher mortality.

**Table 2:** Preoperative, operative, and postoperative univariate analysis of AKI.

**Variables**	**No AKI (*n* = 579)**	**AKI (*n* = 234)**	***P***
**Demographic data**			
Age (year)	61.7 ± 9.0	64.4 ± 8.3	** <0.001**
Male	347 (59.8)	147 (62.8)	0.418
**Previous history**			
Smoking	187 (32.3)	75 (32.1)	0.946
Hypertension	290 (50.1)	122 (52.1)	0.597
Diabetes mellitus	86 (14.9)	56 (23.9)	**0.002**
COPD	8 (1.4)	5 (2.1)	0.537
Cerebrovascular disease	56 (9.7)	39 (16.7)	**0.005**
Anemia	25 (4.3)	17 (7.3)	0.086
**Kidney and cardiac status**			
Serum creatinine (umol/L)	70.6 (59.4, 82.4)	74.9 (61.8, 84.3)	**0.008**
eGFR (ml/min/1.73m^2^)	93.1 (81.2, 102.9)	88.9 (75.3, 99.0)	** <0.001**
NYHA class I-II	362 (62.5)	129 (55.1)	Ref
NYHA class III-IV	217 (37.5)	105 (44.9)	0.051
Ejection fraction (%)	62.7 (57.9, 64.8)	61.9 (53.6, 65.0)	0.175
PAP (mmHg)	33 (28, 41)	33 (27, 40)	0.849
**Preoperative data**			
Hemoglobin (g/L)	132.2 ± 16.0	129.2 ± 17.1	**0.022**
Albumin (g/L)	39.0 ± 3.9	38.2 ± 3.8	** <0.001**
Hematocrit (%)	38.1 ± 7.0	38.5 ± 6.5	0.732
Interval time (day)	7 (4, 11)	7 (5, 10)	0.723
CAG interval≦7d	316 (54.6)	130 (55.6)	0.800
**Intraoperative data**			
**Operation type**			
Isolated CABG	255 (44.0)	66 (28.2)	** <0.001**
Isolated valve	255 (44.0)	108 (46.2)	
CABG+valve	57 (9.8)	47 (20.1)	
Other types	12 (2.2)	13 (5.5)	
ASA Grading	3 (3, 3)	3 (3, 3)	0.188
CPB	357 (61.7)	174 (74.4)	**0.001**
Operating time (min)	263 (224, 325)	289 (232, 373)	**0.001**
CPB time (min)	138 (111, 174)	151 (120, 203)	** <0.001**
Aortic cross clamp time (min)	101 (74, 130)	110 (80, 148)	0.078
**Blood transfusion**			
Red blood cells	487 (84.1)	200 (85.5)	0.628
Plasma	73 (12.6)	40 (17.1)	0.094
Platelets	324 (56.0)	159 (68.0)	**0.002**
**Fluid balance**			
Blood loss (ml)	600 (500, 800)	700 (500, 1,000)	**0.005**
Ultrafiltration (ml)	2,000 (1,200, 2,500)	2200 (1,500, 3,000)	**0.004**
**Postoperative data**			
In-hospital mortality	10 (1.7)	30 (12.8)	** <0.001**
Length of hospital stay (day)	20 (16, 25)	23 (18, 29)	** <0.001**

*eGFR, estimated glomerular filtration rate (CKD-EPI formula); NYHA, New York Heart Association; PAP, pulmonary artery pressure; CABG, coronary artery bypass grafting; CPB, cardiopulmonary bypass*.

*Bold characters are used to express P-values that are statistically significant (< 0.05)*.

In the multivariate analysis, age, diabetes, CPB, cerebrovascular disease, and baseline creatine level were risk factors of CSA-AKI after adjusting for potential confounders (Table 3). However, time interval ≤ 7days was not associated with the incidence of AKI. The result was the same regardless of whether the time interval was considered as a continuous or a multiple categorical variable, or whether age and Scr were substituted with eGFR (Supplementary Table 1). In an attempt to control for the selection bias related to covariates, the propensity score method was applied. As shown in Table 1, the two groups were well-matched, with no significant differences with respect to the variables. Older age (OR = 1.046, 95%CI: 1.017–1.075), CPB (OR = 3.439, 95%CI: 1.316–8.986), diabetes (OR = 2.522, 95%CI: 1.439–4.417) but not time interval ≤ 7days (OR = 1.045, 95%CI: 0.676–1.615) played an important role in the occurrence of CSA-AKI.

**Table 3:** Multivariable logistic analysis for acute kidney injury in the study patients.

**Variables**	**Unadjusted for Propensity score**^**a**^		**Adjusted for Propensity score**^**a**^	
	**OR (95%CI)**	***P*-value**	**OR (95%CI)**	***P*-value**
Age	1.038 (1.017–1.060)	** <0.001**	1.046 (1.017–1.075)	**0.002**
CPB	3.194 (1.536–6.643)	**0.002**	3.439 (1.316–8.986)	**0.012**
Diabetes mellitus	2.712 (1.736–4.236)	** <0.001**	2.522 (1.439–4.417)	**0.001**
CVD	2.020 (1.255–3.252)	**0.004**	1.672 (0.893–3.217)	0.108
Serum creatine	1.009 (1.001–1.016)	**0.030**	1.008 (0.998–1.019)	0.113
Hemoglobin	0.993 (0.982–1.003)	0.167	0.994 (0.981–1.008)	0.431
Albumin	0.972 (0.929–1.017)	0.226	0.940 (0.883–1.002)	0.054
Operating time	1.000 (0.999–1.001)	0.245	1.000 (0.998–1.002)	0.729
Platelet transfusion	0.870 (0.444–1.704)	0.685	0.580 (0.231–1.455)	0.246
Blood loss	1.000 (0.999–1.001)	0.111	1.000 (0.999–1.001)	0.203
Interval ≤ 7days	1.007 (0.713–1.422)	0.967	1.045 (0.676–1.615)	0.844

*CI, confidence interval; CPB, cardiopulmonary bypass; CVD, cerebrovascular disease*.

a *Propensity score developed for the likelihood of receiving CAG interval ≤ 7 days*.

*Bold characters are used to express P-values that are statistically significant (< 0.05)*.

### Subgroup Analysis and Prognosis Analysis

**Figure 1:**
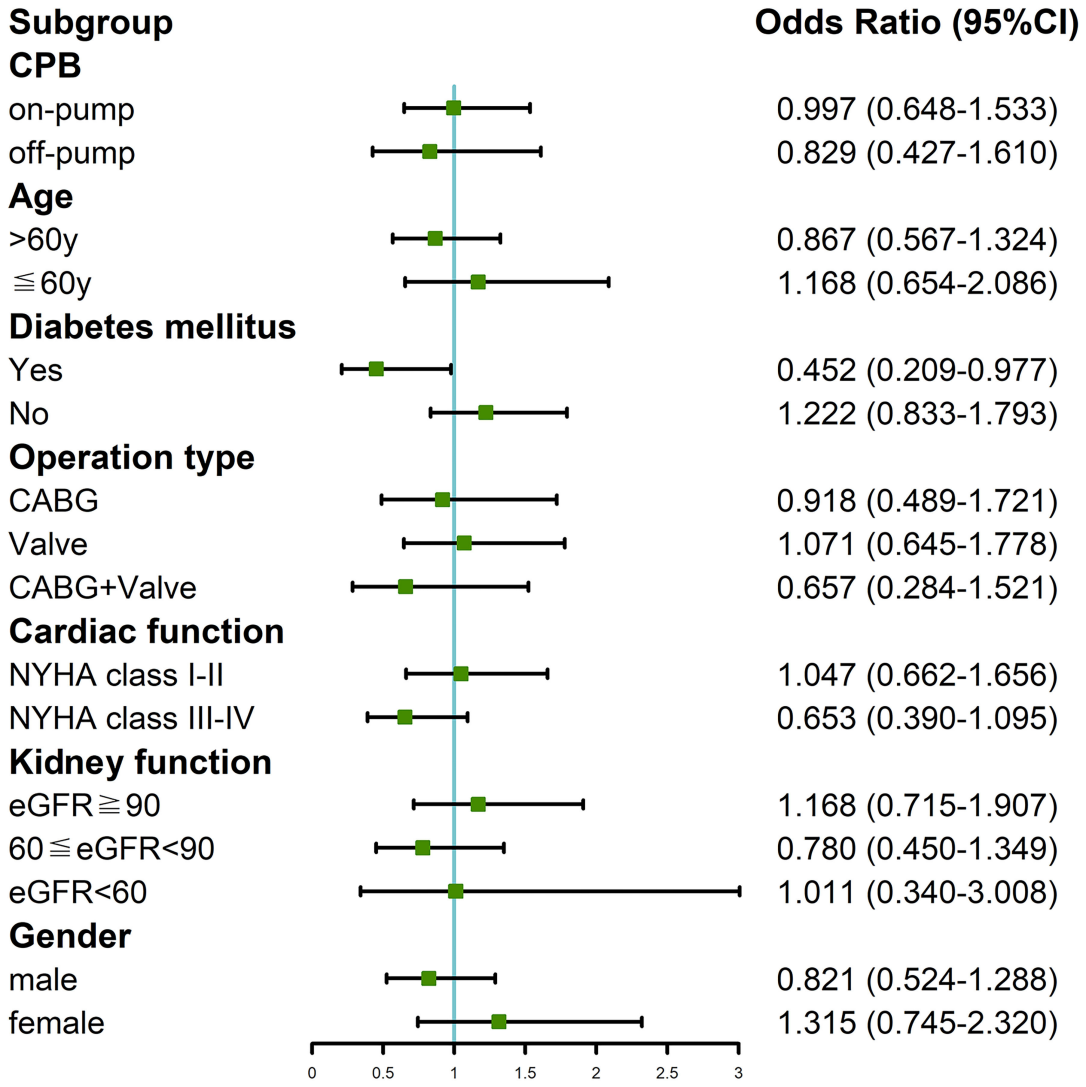
Subgroup analysis of the association between 7-day interval from CAG to cardiac surgery and incidence of CSA-AKI in our cohort study.

We divided the patients into different subgroups according to CPB, age, diabetes, operation type, NYHA classification, eGFR, and gender to probe the potential association between the interval time and CSA-AKI in specific populations. Performing the surgery 7 days after CAG was merely found to mitigate the risk of AKI in patients with diabetes (OR = 0.452, 95%CI: 0.209–0.977; Figure 1). We analyzed prognosis in order to establish the relationship between 7-day interval and CSA-AKI. Detailed outcomes data were shown in Supplementary Table 2. Undergoing cardiac surgery 7 days after CAG did not reduce the risks of RRT (OR = 0.97, 95%CI: 0.44–2.13) or mortality (OR = 0.54, 95%CI: 0.27–1.09) in the multivariate analysis. Performing the surgery after 7 days was also not associated with the risks of RRT (OR = 0.40, 95%CI: 0.10–1.91) or mortality (OR = 0.45, 95%CI: 0.12–1.62) in the subgroup analysis of diabetic patients.

### Results of Meta-Analysis

**Figure 2:**
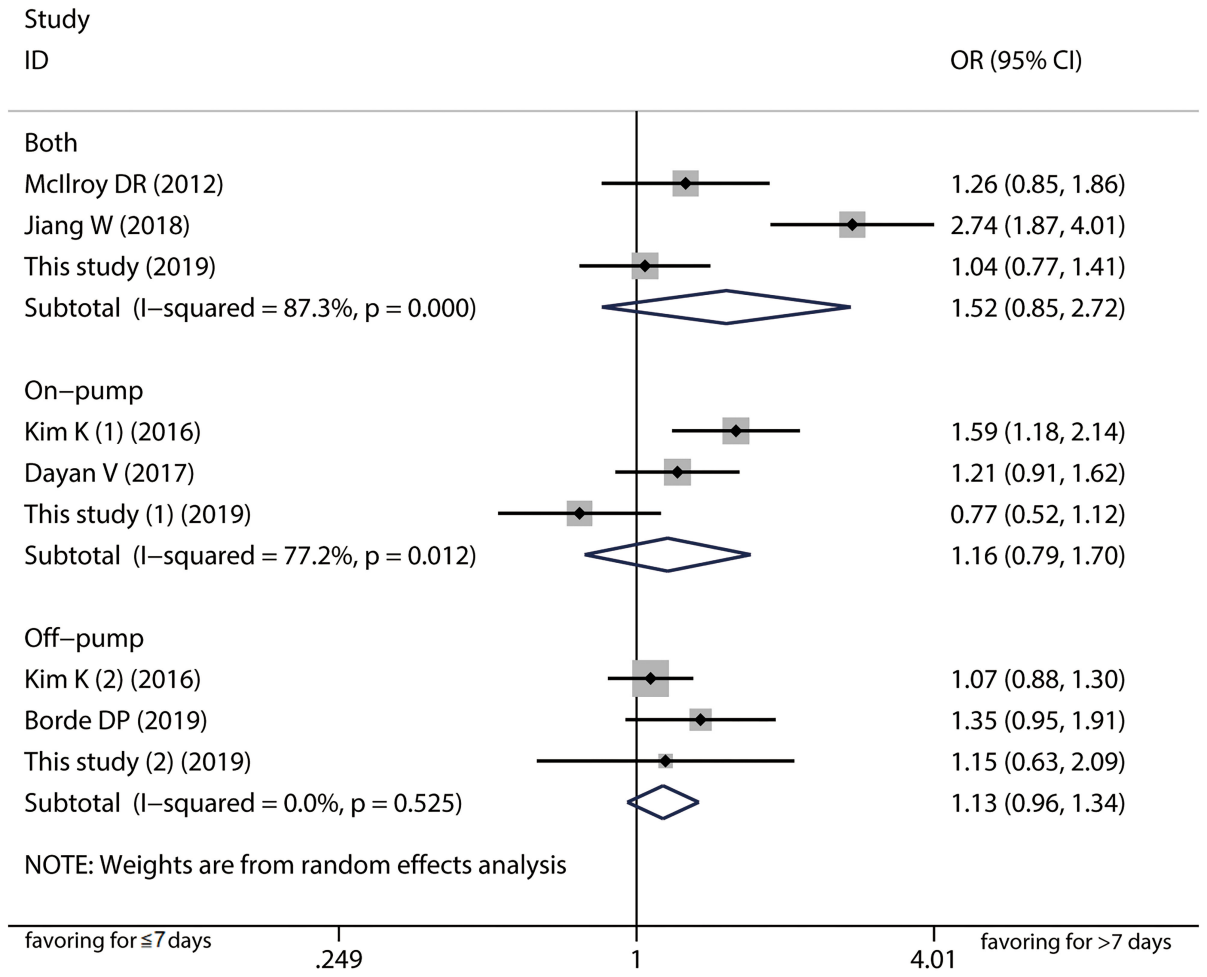
Forest plot comparing ≤ 7-day and >7-day intervals stratified by cardiopulmonary bypass in the meta-analysis. The results indicated AKI risk did not differ between a time interval ≤ 7 days vs. >7 days in the on-pump, off-pump, and both groups.

**Figure 3:**
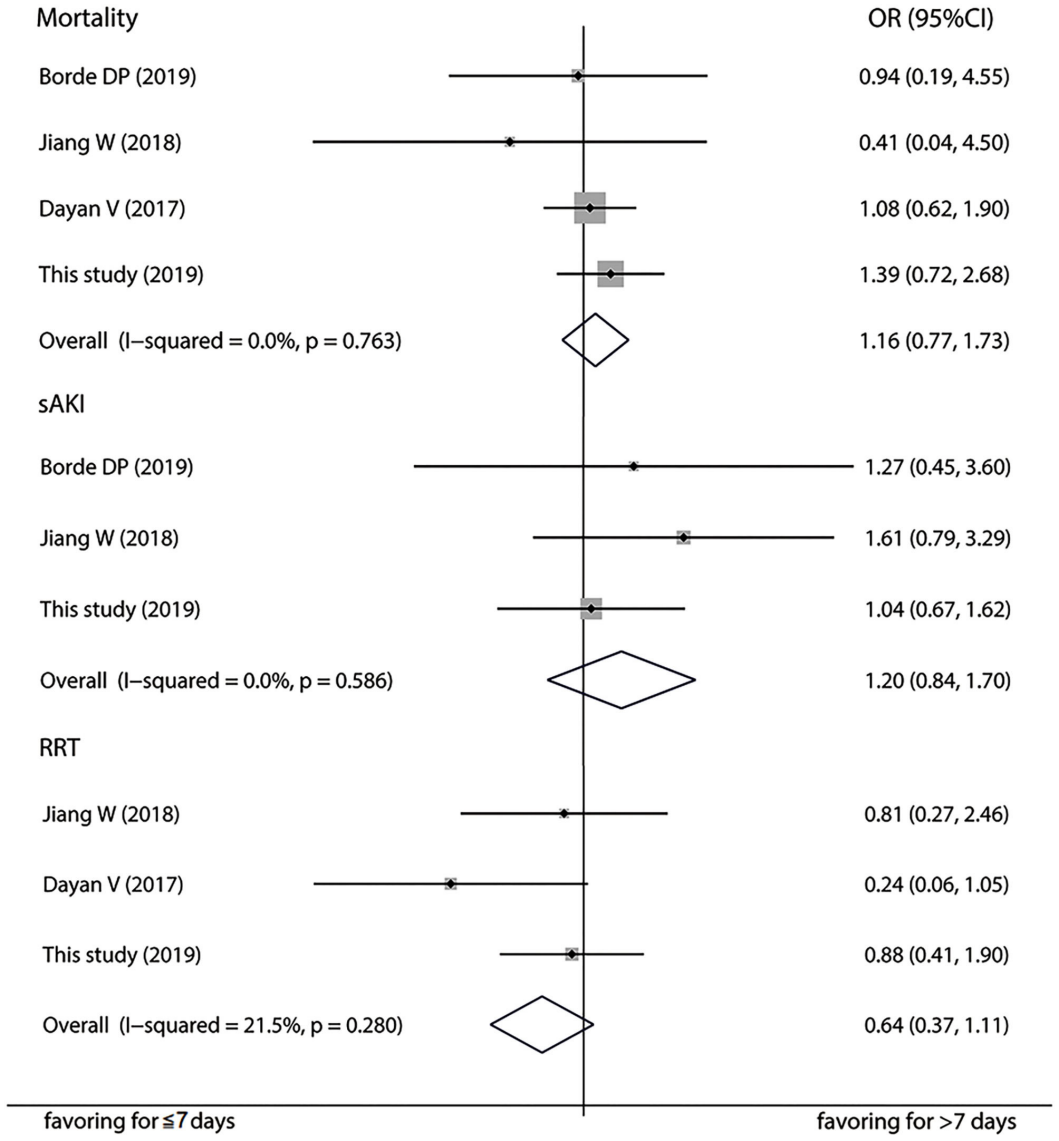
Forest plot comparing ≤ 7-day and >7-day intervals in terms of mortality, severe acute kidney injury, and renal replacement therapy in the meta-analysis.

A total of six papers with scores more than six stars including 6,841 patients were included in the meta-analysis to further verify the association between 7-day interval and the risk of postoperation AKI (17–21) (Supplementary Figure 1). Detailed characteristics of the studies are listed in Table 4, Supplementary Table 2. Pooled analysis of the studies revealed that AKI risk did not differ between a time interval ≤ 7 days vs. >7 days in the on-pump (OR = 1.16, 95%CI: 0.79–1.70; Figure 2) and off-pump (OR = 1.13, 95%CI: 0.96–1.34) subgroups. We also carried out subgroup analyses stratified by ethnicity, AKI definition, and operation type and found not significant associations (Supplementary Table 3). The heterogeneity across studies was low except for the on-pump, Asian and KDIGO subgroups. The sensitivity analysis showed that the results were statistically robust. Moreover, Egger's test revealed no evidence of significant publication bias (*P* = 0.493). The meta-analysis of prognosis exhibited no heterogeneity and no significant differences in mortality, incidence of severe AKI, or risk of RRT (Figure 3).

**Table 4:** Main characteristics of all studies included in the meta-analysis.

**Study**	**Year**	**Country**	**Sample size**	**Male**	**NYHA III-IV**	**Main operation**	**CPB**	**AKI definition**	**AKI incidence**	**Mortality**
McIlroy et al. (17)	2012	USA	644	61.2%	–	Mixed^a^	Both^b^	AKIN	21.9%	3.0%
Kim et al. (18)	2016	Korea	701	73.3%	–	CABG	On-pump	KDIGO	48.9%	1.1%
			1,670	77.0%			Off-pump		36.8%	
Dayan et al. (19)	2017	Uruguay	1,044	54.0%	33.2%	Valve	On-pump	AKIN	27.5%	5.6%
Jiang et al. (20)	2018	China	1,069	62.4%	75.2%	Mixed^a^	Both^b^	KDIGO	38.5%	0.3%
Borde et al. (21)	2019	India	900	78.7%	54.1%	CABG	Off-pump	KDIGO	23.8%	1.0%
This study	2019	China	531	54.4%	43.9%	Mixed^a^	On-pump	KDIGO	32.8%	4.3%
			282	72.3%	31.6%		Off-pump		21.3%	6.0%

*NYHA, New York Heart Association; CPB, cardiopulmonary bypass; AKI, acute kidney injury; CABG, coronary artery bypass grafting; AKIN, Acute Kidney Injury Network; KDIGO, Kidney Disease: Improving Global Outcomes*.

a *Mixed: CABG, Valve and CABG+Valve*.

b *Both: On-pump and Off-pump*.

## Discussion

AKI has been identified as a common complication of cardiac surgery (22). Even less-severe AKI is associated with long in-hospital stay and increased mortality, which imposes a considerable burden on healthcare systems (23). As there is a lack of therapeutic methods to treat CSA-AK, in the past decade attention has shifted from treatment to optimization of perioperative management and avoidance of risk factors. Recently, some researchers made a point that the time interval from CAG to cardiac surgery might be an interventional risk factor for CSA-AKI. For example, Kramer et al. enrolled 668 non-emergent adult cardiac surgical cases and suggested discharge and readmission as a potential strategy to reduce the postoperative incidence of AKI (24). The main evidence supporting for this conclusion is that contrast media may bring about the kidney injury and the underlying pathophysiological mechanisms include a deleterious reduction in renal arteriolar blood flow and glomerular filtration rate, as well as the direct toxicity to tubular epithelial cells (25). The CAG procedure likely induces a pre-injury state in the kidney which can be described as acute kidney stress, such that subsequent cardiac surgery can accelerate the development of AKI. However, in the present retrospective cohort study, we observed that 7-day interval between CAG and cardiac surgery was not a risk factor for the postoperative occurrence of AKI.

Consistent with the findings of our study, a number of previous studies reported similar results. An investigation of 644 patients reported no association between the contrast-to-surgery time interval and occurrence of CSA-AKI, even after increasing the postoperative period over which AKI was defined or including only patients undergoing elective surgery. Borde DP and colleagues analyzed 900 patients who underwent off-pump coronary artery bypass surgery (CABG) after contrast exposure and suggested that performing the surgery within 7 days did not increase the risk of post-operative AKI compared to patients who underwent the operation after 7 days. The first possible explanation is that the influence of CM on kidney function is exaggerated (26). A meta-analysis of 13 studies involving 25,950 patients showed no significant difference in AKI risk between patients with and those without exposure to CM (OR = 0.79, 95%CI = 0.62–1.02) (27). A nationwide analysis of data for about 6 million patients in the U.S. demonstrated AKI rates were nearly identical no matter patients received CM or not, which proved that risk for AKI attributable to CM was modest (28). Secondly, non-nephrologists have become increasingly aware of renal adverse events associated with the use of contrast agents and monitor creatinine frequently, which could to some extent influence the determination of surgery time and reduce the incidence of CSA-AKI. In addition, the use of iso-osmolar CM and advances in CAG should be taken into consideration. In this context, it is not surprising that AKI risk was not increased in cardiac surgical patients who accepted a same-day coronary angiography (29).

It is widely shared that several risk factors acting at pre-, intra-, and postoperative levels play a role in the occurrence of CSA-AKI. In our study, we found age, CPB, diabetes, cerebrovascular disease, and basal renal function but not time interval were contributing factors of CSA-AKI in the multivariate logistic regression analysis. This is consistent with the risk variables reported in previous studies, which emphasizes the multifactorial etiology of CSA-AKI and the need for a comprehensive assessment to establish AKI risk in each patient (4). It should be noted that several previous studies with positive results existed latent selection bias; for instance, some studies included critically ill patients undergoing emergency operation who were susceptible to CSA-AKI (9). Additionally, in some studies the baseline characteristics of patients in the long and short time interval cohorts were not comparable. The exact association between the time interval and CSA-AKI might be misestimated or not properly detected. Given the above, propensity score matching method was adopted to confirm that a shorter time interval did not contribute to a reduced incidence of CSA-AKI. However, age, diabetes, and CPB were found to be independent predictors of CSA-AKI. It is well-known that AKI is often a continuum of kidney injury rather than a single-hit. The CPB procedure may disrupt hemodynamics by ischemia-reperfusion injury, resulting in low cardiac output and hemodilution (4, 30). Senility and diabetes also aggravate renal microvascular dysfunction (31, 32). These risk factors, which can be considered as first hits, predispose the kidney to further injury, with subsequent cardiac surgery being the second hit that causes further deterioration of renal function rather than recovery. In order to clarify the potential correlation between the time interval and CSA-AKI in some specific populations, we conducted subgroup analysis and perceived that undergoing cardiac surgery after 7 days mitigated the risk of AKI merely in patients with diabetes. This finding was actually in agreement with the results of multivariate analysis. We speculate that shortening the time interval worsens renal function, which increases the risk of AKI; preexisting diabetes increases susceptibility to rather than mediates CSA-AKI (33). Finally, the meta-analysis stratified by ethnicity, AKI definition, CPB, and operation type supported the results of our single center study. Notably, heterogeneity was still observed in some subgroups. Our study population had the lowest incidence of CSA-AKI among published studies; differences in perioperative treatment strategies, proportions of operation types, and skill levels in the operation and in nursing across studies could account for this variability. Small sample sizes in the published studies prevented us from further examining the sources of heterogeneity.

Clinicians often focus more on the prognosis of a disease. CSA-AKI remains an important cause of patient morbidity and mortality, which bring it into limelight. A series of studies have shown that postoperative AKI has a moderately poor prognosis (34, 35). In the current study, in-hospital mortality was significantly higher in patients with CSA-AKI than in those without kidney injury. Further analysis indicated that 7-day interval was unrelated to hemodialysis rate and mortality, even in diabetic patients. This result appeared to be contradictory with the interrelation between the time interval and CSA-AKI incidence. We suppose that the correlation does not actually exist, or that the time interval merely affects the incidence of stage 1 AKI, which has a relatively favorable prognosis in most cases. To monitor creatinine level, discontinue nephrotoxic drugs, and stabilize hemodynamics are crucial measures for stage 1 AKI and renal function generally recover without invasive interventions (36, 37). Previous studies uncovered that the nominal increments in creatinine levels used to define AKI were observed in patients exposed to CM (33). As a consequence, a shorter time interval from CAG to cardiac surgery might give rise to a slight elevation in serum creatinine without affecting prognosis. Since there were relatively few cases of RRT and death, we further conducted a meta-analysis of published studies, and the pooled ORs validated our results that the time interval was not an essential factor in the occurrence of severe AKI, RRT, and death.

According to the results of this investigation, delaying cardiac surgery for 7 days after CAG to avoid the development of CSA-AKI is unwarranted. In clinical practice, clinicians should instead focus on the patient profile including comorbidities as well as the surgical procedure. In most patients, contrast-associated acute kidney injury may be transient and reversible in nature. For patients at increased risk of AKI and those with impending AKI, the decision of delaying the surgery should be made after a comprehensive evaluation. Likewise, shortening the time interval should be weighed against the expected benefit. Unfortunately, we do not have any effective tool to help choose the timing of CAG before the surgery so far. In other words, it is unable to obtain an optimal time interval on the basis of our results. This is the most important limitation of this study. It is worth mentioning that scientists have identified several novel biomarkers (e.g., NGAL, KIM 1, IL-18, TIMP2^*^IGFBP7, cystatin C, and GST) released from the kidney during injury or filtered by the kidneys. These biomarkers offer several theoretical advantages over serum creatinine, which are in increasing use to determine the nature of kidney injury, and consequently assess the result of therapy and improve patient management (38, 39). Thus, a well-designed prediction model based on clinical data and biomarkers may be a promising solution. Our study also had several other limitations. Firstly, we included all cardiac surgery patients regardless of the type and mode of surgery, which made the conclusions more applicable to the general population. However, individual differences were neglected. The analyses with refined and detailed evaluation parameters in patients of specific surgical types should be conducted in further large sample size study. Secondly, data on creatinine in the time interval between CAG and surgery were missing for some patients, making it difficult to distinguish between contrast-induced AKI and CSA-AKI in these patients. Thirdly, data of detailed dose of CM, postoperative fluid infusion and some other immeasurable variables were not collected, which might confound the results. We could not rule out the possibility that negative results were due to a type II error. Finally, this was a single center study with a retrospective design. Although we performed subgroup and propensity score analyses as well as meta-analysis to confirm our results, the limited sample size and heterogeneity could undermine the robustness of results. A further large prospective, multicentric, randomized, controlled study is still needed to validate our findings.

## Conclusion

To sum up, undergoing cardiac surgery within 7 days after CAG was not associated with an increased risk of CSA-AKI or worse prognosis. The optimal timing of CAG before surgery should be weighted with reference to the severity of cardiovascular disease and risk factors for CSA-AKI in patients who are susceptible to acute kidney injury. To carry out a well-designed prospective study with large sample size and establish a practical prediction model based on clinical indices and novel renal biomarkers are worthwhile directions in the future.

## Data Availability Statement

The raw data supporting the conclusions of this article will be made available by the authors, without undue reservation.

## Author Contributions

KL, HM, and CX: project design. ML and LL: data collection. KL and BW: statistic analysis. XX and YG: supervision and validation. KL, ML, and HM: writing and revision. All authors: read and approved the final manuscript.

## Conflict of Interest

The authors declare that the research was conducted in the absence of any commercial or financial relationships that could be construed as a potential conflict of interest.

## Supplementary Material

Supplementary Figure 1 Flow diagram of study selection for the meta-analysis.

Supplementary Table 1 Multivariable logistic analysis for acute kidney injury in the study patients in different models.

Supplementary Table 2 Detailed data of all studies included in the meta-analysis.

Supplementary Table 3 Subgroup analyses of Meta-analysis.
