# Supplementary figures and images for: The Effect of Coronary Angiography Timing on Cardiac Surgery Associated Acute Kidney Injury Incidence and Prognosis
Source: Front Med (Lausanne). 2021 Apr 15;8:619210. doi: 10.3389/fmed.2021.619210 (PMC8081843; doi:10.3389/fmed.2021.619210)

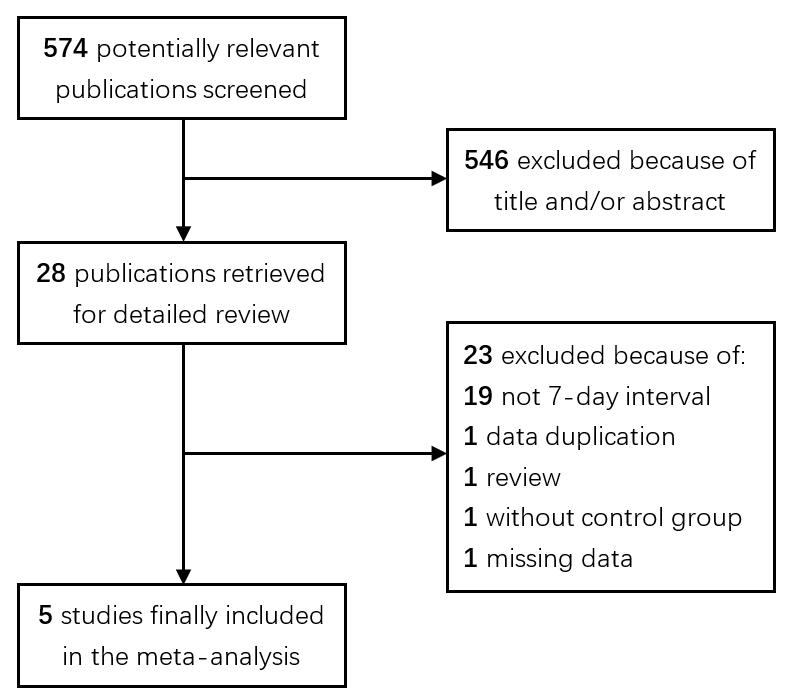

Supplement: Supplementary Figure 1 — Flow diagram of study selection for the meta-analysis. [file Image_1.TIF]
