# Supplementary material for: The Effect of Coronary Angiography Timing on Cardiac Surgery Associated Acute Kidney Injury Incidence and Prognosis
Source: Front Med (Lausanne). 2021 Apr 15;8:619210. doi: 10.3389/fmed.2021.619210 (PMC8081843; doi:10.3389/fmed.2021.619210)
Supplement: Supplementary Table 1 — Multivariable logistic analysis for acute kidney injury in the study patients in different models. [file Table_1.DOCX]

| **Supplementary Table 1. Multivariable logistic analysis for acute kidney injury in the study patients in different models** | | | | | | | | |
| --- | --- | --- | --- | --- | --- | --- | --- | --- |
| **Variables** | **Model 1^a^** | |  | **Model 2^b^** | |  | **Model 3^c^** | |
|  | **OR (95%CI)** | **P value** |  | **OR (95%CI)** | **P value** |  | **OR (95%CI)** | **P value** |
| **Age** | 1.038 (1.016-1.059) | **0.001** |  | 1.038 (1.017-1.060) | **<0.001** |  | - | - |
| **CPB** | 3.331 (1.596-6.952) | **0.001** |  | 3.487 (1.658-7.333) | **0.001** |  | 2.663 (1.300-5.452) | **0.007** |
| **Diabetes mellitus** | 2.717 (1.741-4.240) | **<0.001** |  | 2.701 (1.727-4.225) | **<0.001** |  | 2.626 (1.691-4.077) | **<0.001** |
| **CVD** | 2.020 (1.255-3.251) | **0.004** |  | 2.058 (1.276-3.319) | **0.003** |  | 2.048 (1.277-3.283) | **0.003** |
| **Serum creatine** | 1.008 (1.001-1.016) | **0.034** |  | 1.008 (1.001-.1016) | **0.034** |  | - | - |
| **eGFR** | - | - |  | - | - |  | 0.988 (0.979-0.996) | **0.006** |
| **Hemoglobin** | 0.992 (0.982-1.003) | 0.161 |  | 0.993 (0.982-1.004) | 0.200 |  | 0.992 (0.982-1.002) | 0.132 |
| **Albumin** | 0.973 (0.930-1.019) | 0.246 |  | 0.972 (0.929-1.018) | 0.232 |  | 0.962 (0.920-1.006) | 0.092 |
| **Operating time** | 1.001 (0.999-1.002) | 0.280 |  | 1.000 (0.999-1.002) | 0.297 |  | 1.000 (0.999-1.002) | 0.185 |
| **Platelet transfusion** | 0.875 (0.446-1.718) | 0.698 |  | 0.867 (0.439-1.711) | 0.680 |  | 0.906 (0.467-1.759) | 0.771 |
| **Blood loss** | 1.000 (0.999-1.001) | 0.117 |  | 1.000 (0.999-1.001) | 0.098 |  | 1.000 (0.999-1.001) | 0.115 |
| **Interval** ≤ **7days** | - | - |  | - | - |  | 0.974 (0.687-1.271) | 0.880 |
| **Interval time** | 1.012 (0.982-1.042) | 0.450 |  | - | - |  | - | - |
| **> 7days** | - | - |  | Reference | - |  | - | - |
| **6-7 days** | - | - |  | 1.246 (0.818-1.899) | 0.305 |  | - | - |
| **4-5 days** | - | - |  | 1.229 (0.712-2.120) | 0.459 |  | - | - |
| **0-3 days** | - | - |  | 0.988 (0.813-1.190) | 0.411 |  | - | - |
| CI confidence interval, CPB cardiopulmonary bypass, CVD cerebrovascular disease, eGFR estimated glomerular filtration rate (CKD-EPI formula)  ^a^Model 1: the time interval was incorporated into the regression model as a continuous variable.  ^b^Model 2: the time interval was incorporated into the regression model as a multiple categorical variable.  ^c^Model 3: the model was established after adjusting for CPB, diabetes mellitus, CVD, eGFR, hemoglobin, albumin, operating time, platelet transfusion, blood loss and Interval ≤ 7days. | | | | | | | | |
