# Supplementary material for: The Effect of Coronary Angiography Timing on Cardiac Surgery Associated Acute Kidney Injury Incidence and Prognosis
Source: Front Med (Lausanne). 2021 Apr 15;8:619210. doi: 10.3389/fmed.2021.619210 (PMC8081843; doi:10.3389/fmed.2021.619210)
Supplement: Supplementary Table 2 — Detailed data of all studies included in the meta-analysis. [file Table_2.DOCX]

| **Supplementary Table 2. Detailed data of all studies included in the meta-analysis** | | | | | | | | | | | | |
| --- | --- | --- | --- | --- | --- | --- | --- | --- | --- | --- | --- | --- |
| **Author** | **Age^a^** | **CAG interval ≤7 days** | | | | |  | **CAG interval >7 days** | | | | |
|  |  | **Total** | **AKI** | **SAKI^b^** | **Mortality** | **RRT** |  | **Total** | **AKI** | **SAKI^b^** | **Mortality** | **RRT** |
| McIlroy DR [17] | 68 (11) / 69 (13) | 398 | 93 | - | - | - |  | 246 | 48 | - | - | - |
| Kim K [18] | 63.5 (9.0) | 341 | 190 | - | - | - |  | 360 | 159 | - | - | - |
|  | 63.7 (9.3) | 857 | 322 | - | - | - |  | 813 | 293 | - | - | - |
| Dayan V [19] | 69.1 (11.3) / 65.3 (11.5) | 325 | 98 | - | 19 | 2 |  | 719 | 189 | - | 39 | 18 |
| Jiang W [20] | 61.3 (8.1) / 63.9 (8.3) | 888 | 374 | 69 | 2 | 16 |  | 181 | 38 | 9 | 1 | 4 |
| Borde DP [21] | 60 (10) / 60 (9) | 210 | 59 | 5 | 2 | - |  | 690 | 155 | 13 | 7 | - |
| This study | 60.7 (8.7) / 62.6 (7.5) | 354 | 109 | 49 | 25 | 14 |  | 177 | 65 | 39 | 15 | 13 |
|  | 64.8 (8.7) / 64.6 (9.7) | 92 | 21 |  |  |  |  | 190 | 39 |  |  |  |
| CAG coronary angiography, AKI acute kidney injury, SAKI severe acute kidney injury, RRT renal replacement therapy  ^a^Age: ≤7 / >7, the values are expressed as the mean (SD)  ^b^SAKI: AKI KDIGO stage 2-3 | | | | | | | | | | | | |
