# Supplementary material for: The Effect of Coronary Angiography Timing on Cardiac Surgery Associated Acute Kidney Injury Incidence and Prognosis
Source: Front Med (Lausanne). 2021 Apr 15;8:619210. doi: 10.3389/fmed.2021.619210 (PMC8081843; doi:10.3389/fmed.2021.619210)
Supplement: Supplementary Table 3 — Subgroup analyses of Meta-analysis. [file Table_3.DOCX]

| **Supplementary Table 3. Subgroup analyses of Meta-analysis** | | | | | |
| --- | --- | --- | --- | --- | --- |
| **Subgroup** | **N** | **P_h_^a^** | **OR** | **95%CI** | **P** |
| **CPB** |  |  |  |  |  |
| On-pump | 3 | 0.012 | 1.16 | 0.79-1.70 | 0.461 |
| Off-pump | 3 | 0.525 | 1.13 | 0.96-1.34 | 0.144 |
| Both | 3 | 0.000 | 1.52 | 0.85-2.72 | 0.157 |
| **Ethnicity** |  |  |  |  |  |
| Asian | 6 | 0.000 | 1.33 | 0.96-1.84 | 0.084 |
| American | 2 | 0.878 | 1.23 | 0.97-1.55 | 0.085 |
| **Definition** |  |  |  |  |  |
| KDIGO | 6 | 0.000 | 1.33 | 0.96-1.84 | 0.084 |
| AKIN | 2 | 0.878 | 1.23 | 0.97-1.55 | 0.085 |
| **Operation** |  |  |  |  |  |
| CABG | 4 | 0.122 | 1.24 | 0.99-1.55 | 0.060 |
| Valve | 2 | 0.216 | 1.07 | 0.77-1.49 | 0.686 |
| CPB cardiopulmonary bypass, CABG coronary artery bypass grafting  ^a^P_h_ P value for heterogeneity test | | | | | |
